# CD8+ T cells in Hashimoto’s thyroiditis-associated papillary thyroid carcinoma

**DOI:** 10.1530/ETJ-25-0365

**Published:** 2026-06-09

**Authors:** Lirong Wang, Lin Zhang, Jiawen Chen, Dan Wang, Yutong Zhang, Lei Sun, Wenxiu Su, Miao Li, Qi Zhou, Juan Wang, Jue Jiang

**Affiliations:** ^1^ Department of Ultrasound, The Second Affiliated Hospital of Xi’an Jiaotong University, Xi’an, China; ^2^ Department of Ultrasound, The Second Affiliated Hospital of Zhejiang University School of Medicine, Hangzhou, China; ^3^ Department of Otorhinolaryngology Head and Neck Surgery, The Second Affiliated Hospital of Xi’an Jiaotong University, Xi’an, China; ^4^ Department of Pathology, The Second Affiliated Hospital of Xi’an Jiaotong University, Xi’an, China

**Keywords:** papillary thyroid carcinoma, Hashimoto’s thyroiditis, CD8+ T cells

## Abstract

Papillary thyroid carcinoma (PTC) is the most common thyroid malignancy and frequently coexists with Hashimoto’s thyroiditis (HT). Clinical observations indicate that HT-PTC tends to display less aggressive clinicopathological features; however, the immune characteristics underlying this phenomenon remain incompletely defined. In this study, we combined transcriptomic analysis with experimental validation to investigate CD8+ T cell features in HT-PTC. Analysis of TCGA data showed increased CD8+ T cell infiltration in HT-PTC compared with non-HT-PTC. In surgically resected specimens from our center, primary CD8+ T cells derived from HT-PTC were further characterized. Tumor-derived CD8+ T cells maintained cytokine secretion capacity and showed lower expression of TIM-3 and LAG-3, together with higher Granzyme B levels. In coculture assays, these cells were associated with reduced proliferation and migration of thyroid cancer cells. Overall, CD8+ T cells in HT-PTC exhibited no pronounced exhaustion-associated feature pattern and retained functional activity. This immune profile may be linked to the relatively less aggressive behavior observed in HT-PTC and provides a biological reference for future immune-informed risk stratification strategies.

## Introduction

Papillary thyroid carcinoma (PTC) is the most common endocrine malignancy, and its global incidence continues to rise (1). By 2024, PTC ranked as the seventh most frequently diagnosed cancer worldwide, with an estimated 821,173 new cases (2). Although PTC often presents with a high rate of cervical lymph node metastasis (up to 64%), its overall prognosis remains excellent, with a 10-year survival rate exceeding 95% (3, 4). This discrepancy highlights the clinical need to better distinguish aggressive from indolent subtypes to support individualized management and reduce the risk of overtreatment in low-risk patients.

Hashimoto’s thyroiditis (HT), a prevalent autoimmune thyroid disorder, has been frequently associated with PTC, with a coexistence rate ranging from 20 to 50% (5). Numerous clinical studies have reported that PTC coexisting with HT exhibits less aggressive characteristics than PTC alone, including smaller tumor size, lower rates of lymph node metastasis and extrathyroidal extension, fewer distant metastases, and improved long-term outcomes (6, 7, 8). These consistent findings suggest that the immune microenvironment associated with HT may be associated with a less aggressive tumor phenotype in PTC. However, the underlying immunological mechanisms remain largely undefined.

Increasing evidence highlights the crucial role of the tumor immune microenvironment in thyroid carcinogenesis and progression. Single-cell transcriptomic profiling of HT lesions has revealed an immune infiltrate dominated by T and NK cells, with particularly abundant CD8+ T cells (9). Similarly, dense infiltration of CD8+ T cells recognizing thyroid-specific antigens (such as TPO and Tg) has been observed in PTC tissues, suggesting an association with antitumor activity (10). CD8+ T cells may, therefore, indicate an immunologic link between HT and the less invasive behavior of PTC. Nevertheless, the antitumor efficacy of CD8+ T cells depends critically on their functional state rather than their mere presence. In chronic antigenic stimulation, these cells can acquire exhaustion-like phenotypes characterized by diminished cytotoxicity and sustained expression of inhibitory receptors such as PD-1 and Tim-3 (11). Severson *et al.* (12) identified PD-1+Tim-3+CD8+ T cell subsets in metastatic differentiated thyroid carcinoma that retained proliferative capacity but lacked perforin expression, suggesting functional impairment associated with exhaustion-like phenotypes.

Based on these observations, we hypothesize that the HT microenvironment may preserve CD8+ T cells activation or modulate exhaustion-like phenotypes, thereby contributing to a less aggressive tumor phenotype in HT-PTC. To address this hypothesis, we performed an integrated investigation combining bioinformatic analyses and experimental validation. Transcriptomic data from TCGA were first analyzed to evaluate CD8+ T cell infiltration and associated clinicopathological features in HT-PTC and non-HT-PTC. Primary CD8+ T cells were then isolated from surgically resected specimens to assess exhaustion-associated features, cytokine secretion, and cytotoxic capacity *in vitro*. This study aims to systematically characterize the functional state of CD8+ T cells in HT-PTC and explore its biological relevance to the less aggressive clinicopathological features observed in this subgroup, providing a potential immune-based perspective for understanding risk heterogeneity in PTC.

## Materials and methods

### Analysis of immune cell infiltration

RNA expression data and corresponding clinical information for PTC patients were retrieved from The Cancer Genome Atlas (TCGA) database. Patients were divided into two groups according to the presence or absence of HT (HT-PTC and non-HT-PTC). To minimize potential confounding from intrinsically aggressive histological variants, only classical PTC and its follicular variant were included in the analysis. In TCGA-based samples, HT status was determined according to the pathological annotation of lymphocytic thyroiditis, which is commonly used as the histopathological correlate of HT. The exclusion criteria were as follows: (i) patients with other types of thyroid tumors and (ii) patients without thyroid disease assessment data. Detailed data processing steps and corresponding R code can be found in Supplementary Fig. S1 and the Appendix (see section on Supplementary materials given at the end of the article). When a gene was recorded multiple times, the average expression level was used. The CIBERSORT algorithm (13) was applied to analyze the normalized gene expression data from the HT and non-HT groups and estimate the relative infiltration percentages of CD8+ T cells.

### Clinical tissue samples

With approval from the Ethics Committee of the Second Affiliated Hospital of Xi’an Jiaotong University (2022259), this study included 71 patients diagnosed with PTC between May 2022 and December 2022 (19 HT-PTC and 52 non-HT-PTC). Only classical PTC and its follicular variant were included, and recurrence risk was stratified according to the ATA 2025 guidelines (14). Detailed information is provided in Supplementary Table S1 and Supplementary Table S7. All patients signed informed consent forms prior to surgery and had not received any other treatments. In this setting, HT was defined by histopathological lymphocytic thyroiditis together with preoperative positivity for thyroid autoantibodies (TPOAb and/or TgAb). Postoperatively, the tumor and peritumoral tissues were immediately collected. One portion was transferred to liquid nitrogen and stored at −80°C, while another portion was rinsed with PBS and fixed in 4% paraformaldehyde for paraffin embedding.

Among these patients, 17 cases (6 HT-PTC and 11 non-HT-PTC) with larger tumors (≥1 cm) were selected to ensure sufficient tissue yield for primary CD8+ T cell isolation, subsequent *in vitro* functional assays, and flow cytometric analyses. Detailed clinical information is provided in Supplementary Table S2. The tumor and peritumoral tissues from these patients were rinsed with PBS, placed in precooled tissue preservation solution (Bio-channel,China, BC-CFM-03), stored at 4°C, and processed into single-cell suspensions within three days.

In addition, nodular goiter and simple HT tissues were included as benign disease controls to provide a nonmalignant reference for immune phenotyping. For multiplex immunofluorescence analysis, a representative subset of samples was selected from each group (*n* = 6 per group). Nodular goiter was defined as benign nodular hyperplasia of the thyroid without histological evidence of malignancy or lymphocytic thyroiditis. Simple HT was defined as diffuse lymphocytic thyroiditis without malignant lesions and with positive serum TPOAb and/or TgAb.

### Total RNA extraction and real-time PCR (RT-PCR)

Total RNA was extracted from PTC patient tissue samples using Trizol reagent (Life Technologies Corporation, USA). The quality and concentration of RNA were measured at 260/280 nm using a Nanodrop spectrophotometer (Thermo, USA). cDNA was synthesized from total RNA using a reverse transcription kit (TaKaRa, RR036A), and real-time PCR was performed using a real-time PCR kit (TaKaRa, RR820A) according to the manufacturer’s instructions. The expression of the CD8A gene was normalized to the expression level of glyceraldehyde-3-phosphate dehydrogenase (GAPDH).

Primers used for amplification were: CD8A (Fwd: AGACAGTGGAGCTGAAGTGC; Rev: TAGGAGGAAGGTGGGACTGG) and GAPDH (Fwd: GGAGTCCACTGGCGTCTTCA; Rev: GTCATGAGTCCTTCCACGATACC).

### Immunohistochemistry (IHC) staining and multiplex immunofluorescence (mIF) analysis

Paraffin-embedded tissue sections (4 μm) were dewaxed in xylene and rehydrated through a graded ethanol series. Heat-induced antigen retrieval was performed using citrate buffer (pH 6.0). Nonspecific binding was blocked with immunostaining blocking solution (Beyotime, China, P0102), followed by incubation with the primary antibody CD8a (1:400, Servicebio, China, GB115692-100). Staining was visualized using DAB, counterstained with hematoxylin, and observed under a microscope after mounting. Negative controls were prepared by replacing the primary antibody with PBS.

Multiplex immunohistochemistry/immunofluorescence was performed on FFPE tissue sections (4 μm) from HT PTC, non-HT PTC, nodular goiter, and simple HT using the tyramide signal amplification (TSA) system. After deparaffinization, rehydration, antigen retrieval, and endogenous peroxidase blocking, each section underwent four sequential rounds of staining. Each round included serum blocking, primary antibody incubation, secondary antibody application, TSA fluorophore labeling, and antibody stripping. The primary antibodies used were CD8a (Proteintech, China, 66868-1-Ig), TIM3 (Proteintech,11872-1-AP), LAG3 (Proteintech, 29548-1-AP), and Granzyme B (Proteintech, 13588-1-AP). Corresponding TSA reagents were iF440-Tyramide (Servicebio, G1250), iF488-Tyramide (Servicebio, G1231), iF555-Tyramide (Servicebio, G1233), and iF647-Tyramide (Servicebio, G1232). Slides were scanned using a Pannoramic MIDI digital scanner (3DHISTECH, Hungary), and quantification of TIM3+CD8+, LAG3+CD8+, and Granzyme B+CD8+ T cells was performed using the HALO platform (Indica Labs, USA).

### Cytokine detection

The concentrations of human IL-2 (CHE0057-096), IFN-γ (CHE0017-096), and TNF-α (CHE0019-096) were measured using ELISA kits (4A BIOTECH, China) according to the manufacturer’s instructions. Sample concentrations were calculated based on standard curves.

### Cell source and culture

The human PTC cell line TPC-1 (The American Type Culture Collection) was cultured in RPMI-1640 medium (Gibco, USA, 11875093) supplemented with 10% fetal bovine serum (FBS, BI, 04-001-1B) and 1% penicillin–streptomycin (Solarbio, P1400). For experimental groups, CD8+ T cells were isolated from tumors and adjacent tissues of PTC patients with HT, confirmed by pathology and preoperative serological tests showing positive antithyroid antibodies. For control groups, CD8+ T cells were derived from the peripheral blood of PTC patients with HT and healthy individuals. These cells were cultured in RPMI-1640 medium supplemented with 10% heat-inactivated FBS (45°C), 10 ng/mL recombinant IL-2 (PeproTech, USA, 200-02-50UG), and 1% penicillin–streptomycin. All cells were maintained at 37°C in a humidified incubator with 5% CO_2_, with the medium replaced regularly to ensure cell viability.

### Preparation of single-cell suspensions from tissues

Tumor and peritumoral tissues were placed in ice-cold PBS and finely minced with ophthalmic scissors into fragments smaller than 1 mm^3^. The tissue fragments were then incubated in a digestion mixture containing type II collagenase (1 mg/mL, MKbio, China, 9001-12-1) and DNase (0.1 mg/mL, MKbio, 9003-98-9) at 37°C. The mixture was digested in a cell incubator for 6 h, with the culture dish shaken gently every 30 min to ensure even digestion. After incubation, the mixture was filtered through a 70 μm cell strainer, and the filtrate was collected. The cells were centrifuged at 300 ***g*** for 10 min, and the supernatant was discarded. The pellet was washed with precooled HBSS, centrifuged again, and the supernatant was discarded. Finally, the cells were resuspended in PBS containing 2% FBS, and the cell concentration was adjusted to 1 × 10^6^ cells/100 μL.

### Isolation of primary CD8+ T cells

The single-cell suspensions from tissues or PBS-diluted anticoagulated whole blood (1:1 ratio) were carefully layered onto the lymphocyte separation medium (Solarbio, P8610) along the tube wall. The samples were centrifuged at 800 ***g*** for 20 min at room temperature without braking, and the peripheral blood mononuclear cell (PBMC) layer was collected. PBMCs were washed with PBS, resuspended, and filtered through a 70 μm cell strainer. The filtrate was centrifuged at 300 ***g*** for 5 min, and the pellet was resuspended in PBS. The cell concentration was adjusted to 1 × 10^8^ cells/mL. CD8+ T cells were isolated using a CD8+ T Cell Isolation Kit (Biolegend, USA, 480011) based on magnetic bead negative selection, following the manufacturer’s instructions. The purity of the isolated CD8+ T cells was assessed by flow cytometry.

### Functional assay of primary CD8+ T cells

Isolated CD8+ T cells were stimulated at 37°C for 6 h using PMA (50 ng/mL, GLPBIO, USA, GN10444), Ionomycin (1 μg/mL, GLPBIO, GC15148), and BFA (10 μg/mL, GLPBIO, GC17683). After stimulation, the expression of PD-1 (PD1-APC, 4A BIOTECH) on the cell surface and intracellular cytokines IL-2 (IL-2-APC, Elabscience, China), IFN-γ (IFN-γ-APC, 4A BIOTECH), and TNF-α (TNF-α-APC, 4A BIOTECH) was analyzed. Specific protocols followed the instructions provided by the respective manufacturers.

### Activation of primary CD8+ T cells

Primary CD8+ T cells were seeded at a concentration of 1 × 10^6^ cells/mL in six-well plates, with CD3/CD28 T cell activators added at 25 μL/mL (STEMCELL, Canada, 10991). The cells were co-stimulated for three days, with half of the medium replaced by fresh complete medium. Fresh complete medium was added daily. When the cell concentration reached 1 × 10^8^ cells/mL, the cells were transferred to culture flasks for continued cultivation. The medium was partially replaced every 2–3 days. CD8+ T cells in the logarithmic growth phase were selected for coculture experiments with TPC-1 cells.

### Flow cytometry analysis

A single-cell suspension (5 μL and 100 μL/10^6^ cells) was incubated with fluorescence-labeled antibodies (CD3-PE and CD8a-FITC, Elabscience) at 4°C in the dark for 30 min. After staining, the cells were washed twice with PBS and analyzed using a flow cytometer (BD FACSCanto, USA). For each sample, at least 10,000 events were recorded. The gating strategy is shown in Supplementary Fig. S3.

### Coculture of TPC-1 and CD8+ T cells

Expanded CD8+ T cells cultured for 14 days were used as effector cells, and TPC-1 cells cultured for 14 days were used as target cells. Both were adjusted to a concentration of 1 × 10^6^ cells/mL. CD8+ T cells and TPC-1 cells were mixed at effector-to-target (E:T) ratios of 5:1 in 96-well plates. Coculture was performed for 48 h, with duplicate wells for each group. Supernatants were collected at 6, 12, 24, and 48 h, and the concentrations of IFN-γ, IL-2, and TNF-α were measured.

### CCK8 proliferation assay

The CCK8 assay kit (TargetMol, USA, C0005) was used to assess cell proliferation according to the manufacturer’s instructions. The inhibition rate of TPC-1 cells cocultured with CD8+ T cells for 48 h was determined, along with their proliferation rates on days 1, 2, 3, 4, 5, 6, and 7.

### Wound healing assay

TPC-1 cells cocultured with CD8+ T cells were seeded in 12-well plates (1 × 10^5^ cells/well). When the cells reached 80% confluence, a thin ‘wound’ was created using a 200 μL pipette tip. Each group included two replicates. Images were captured every 24 h for 3 days under a microscope, with five images taken per well.

### Transwell migration assay

TPC-1 cells cocultured with CD8+ T cells (1 × 10^5^ cells/200 μL) were suspended in serum-free medium and seeded into the upper chambers of Transwell inserts without Matrigel coating. The lower chambers were filled with complete medium containing 10% FBS as a chemoattractant. The Transwells were incubated at 37°C with 5% CO_2_ for 24 h. After incubation, cells remaining on the upper chamber surface were removed with a cotton swab. The membranes were fixed in 4% paraformaldehyde for 10 min and stained with 0.1% crystal violet for 20 min. Migrated cells on the lower membrane surface were photographed and counted under a microscope.

### Statistical analysis

Statistical analyses were performed using IBM SPSS Statistics 20.0 and R-4.2.0, while GraphPad Prism 9.0.0 was used for plotting. Flow cytometry data were analyzed using FlowJo software, and migration distances in wound healing assays were measured with ImageJ software. For IHC and mIF, three non-overlapping microscopic fields per section were randomly selected and averaged for quantitative analysis. For flow cytometry analyses, data were derived from independent patient samples. For ELISA and coculture assays, each biological sample was tested in triplicate technical replicates. Data following a normal distribution were expressed as mean ± standard deviation (SD), while skewed data were described as median (interquartile range, IQR). Categorical data were presented as frequencies and proportions. Independent sample *t*-tests, Mann–Whitney U tests, chi-square tests, and Fisher’s exact tests were used to compare differences between groups. For three or more groups, one-way ANOVA followed by Tukey’s multiple comparisons test was applied. Migration rates in wound healing assays were analyzed using two-way ANOVA with Holm–Šídák multiple comparisons. All statistical analyses were two-tailed, and a *P*-value <0.05 was considered statistically significant.

## Results

### Clinicopathological features and CD8+ T cell infiltration in HT-PTC and non-HT-PTC based on TCGA analysis

**Figure 1:**
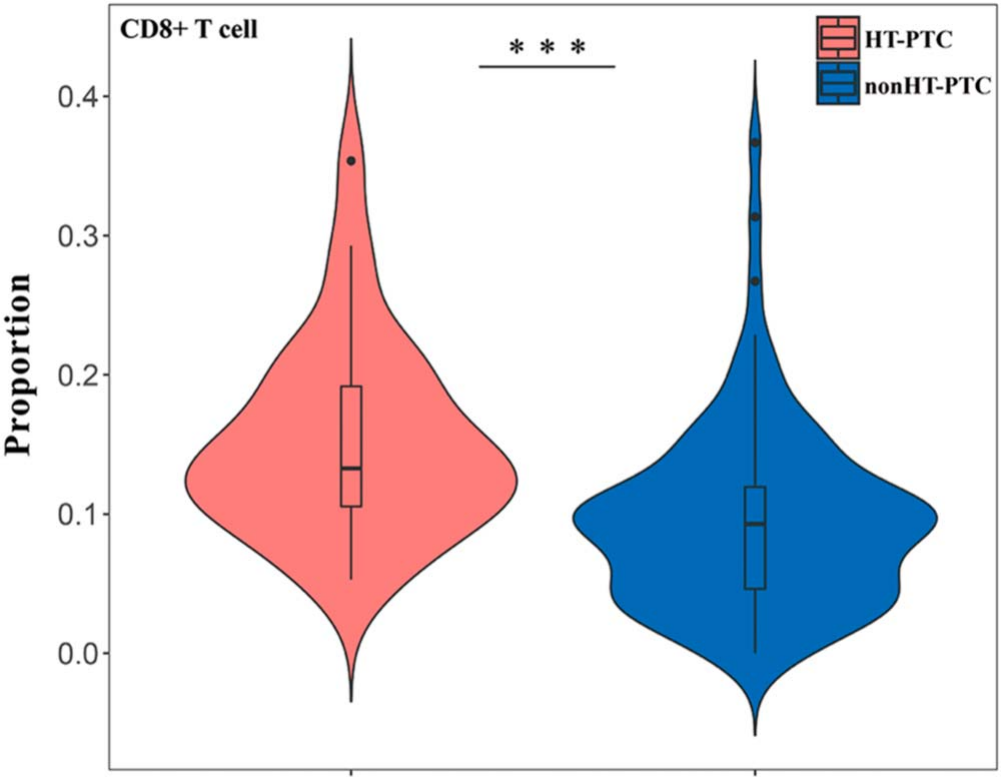
Differences in CD8+ T cell infiltration between HT-PTC and non-HT-PTC. ***P < 0.001.

Compared with non-HT-PTC, HT-PTC exhibited less aggressive clinicopathological features. Specifically, HT-PTC showed a higher proportion of N0 disease (60.4 vs 43.7%, *P* = 0.035), a lower rate of extrathyroidal extension (14.6 vs 33.3%, *P* = 0.010), and a greater proportion of early AJCC stage (I–II: 85.7 vs 67.9%, *P* = 0.012). A higher frequency of multifocal tumors was also observed in HT-PTC (56.3 vs 35.3%, *P* = 0.006), suggesting a more complex but not necessarily more aggressive growth pattern (Table 1). Given these differences in clinical presentation, we next examined whether immune infiltration patterns might be associated with this distinct phenotype. CD8+ T cell infiltration was significantly higher in HT-PTC than in non-HT-PTC (*P* < 0.001; Fig. 1).

**Table 1:** Clinical characteristics of TCGA PTC patients. Statistically significant values (*P<*0.05) are presented in bold.

Characteristics	HT-PTC (*n* = 49)	Non-HT-PTC (*n* = 253)	Statistics	*P*
HT status	Positive	Negative		
Age, years	42.41 ± 14.86	45.26 ± 15.98	1.262	0.251*
Sex			0.614	0.433^†^
Male	10 (20.4)	65 (25.7)		
Female	39 (79.6)	188 (74.3)		
Histological type			1.548	0.213^†^
Classical	37 (75.5)	210 (83.0)		
Follicular	12 (24.5)	43 (17.0)		
Tumor location^§^			1.176	0.759^†^
Left lobe	19 (38.8)	93 (37.2)		
Right lobe	22 (44.9)	112 (44.8)		
Isthmus	1 (2.0)	14 (5.6)		
Bilateral	7 (14.3)	31 (12.4)		
Focus type^§^			7.450	**0.006^†^**
Multifocal	27 (56.3)	89 (35.3)		
Unifocal	21 (43.7)	163 (64.7)		
Extrathyroidal extension^§^			6.699	**0.010^†^**
Yes	7 (14.6)	83 (33.3)		
No	41 (85.4)	166 (66.7)		
T stage^§^			5.808	0.113^‡^
1	16 (32.6)	61 (24.2)		
2	21 (42.9)	91 (36.1)		
3	12 (24.5)	84 (33.3)		
4	0 (0)	16 (6.4)		
N stage^§^			4.435	**0.035^†^**
0	29 (60.4)	97 (43.7)		
1	19 (39.6)	125 (56.3)		
M stage^§^			/	1.000^‡^
0	18 (100)	150 (95.5)		
1	0 (0)	7 (4.6)		
AJCC^§^			6.323	**0.012^†^**
I–II	42 (85.7)	171 (67.9)		
III–IV	7 (14.3)	81 (32.1)		

* Independent samples *t*-test.

^†^ Chi-square test.

^‡^ Fisher’s exact test.

^§^ Some patients had incomplete clinical information; however, their PTC pathology, HT status, and gene expression data were clearly defined, and therefore, they were retained in the analysis.

To further investigate the functional characteristics of CD8+ T cells in this setting, we analyzed surgically resected specimens from patients treated at our center with HT-PTC and non-HT-PTC.

### CD8A expression levels in tumors from HT-PTC and non-HT-PTC groups

**Figure 2:**
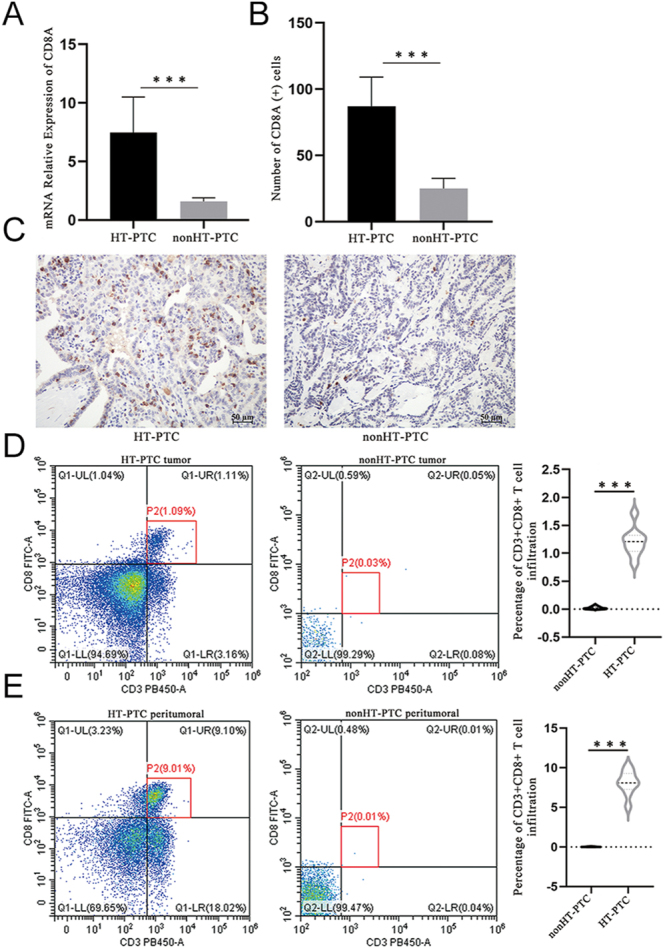
Validation of CD8A expression levels and CD8+ T cell infiltration proportions in HT-PTC and non-HT-PTC. (A) Relative mRNA expression levels of CD8A. (B) Number of CD8A-positive cells identified by IHC (HT-PTC *n* = 19, non-HT-PTC *n* = 52). (C) Representative IHC images. (D) Proportions of CD3+CD8+ T cells in tumor tissues. (E) Proportions of CD3+CD8+ T cells in peritumoral tissues (HT-PTC *n* = 6, non-HT-PTC *n* = 11). ***P < 0.001.

CD8A, a surface marker of CD8+T cells, exhibited significantly elevated mRNA expression in tumoral tissues from the HT-PTC group compared with the non-HT-PTC group (Fig. 2A). Immunohistochemical staining further revealed a significantly higher number of CD8A-positive cells in the HT-PTC group than in the non-HT-PTC group (Fig. 2B and C). Flow cytometry analysis demonstrated that the relative infiltration proportions of CD3+CD8+ T cells were significantly higher in both tumor and peritumoral tissues of HT-PTC compared with those of non-HT-PTC (Fig. 2D and E).

### CD8+ T cell infiltration and associated cytokine levels in HT-PTC tumor and peritumoral tissues

**Figure 3:**
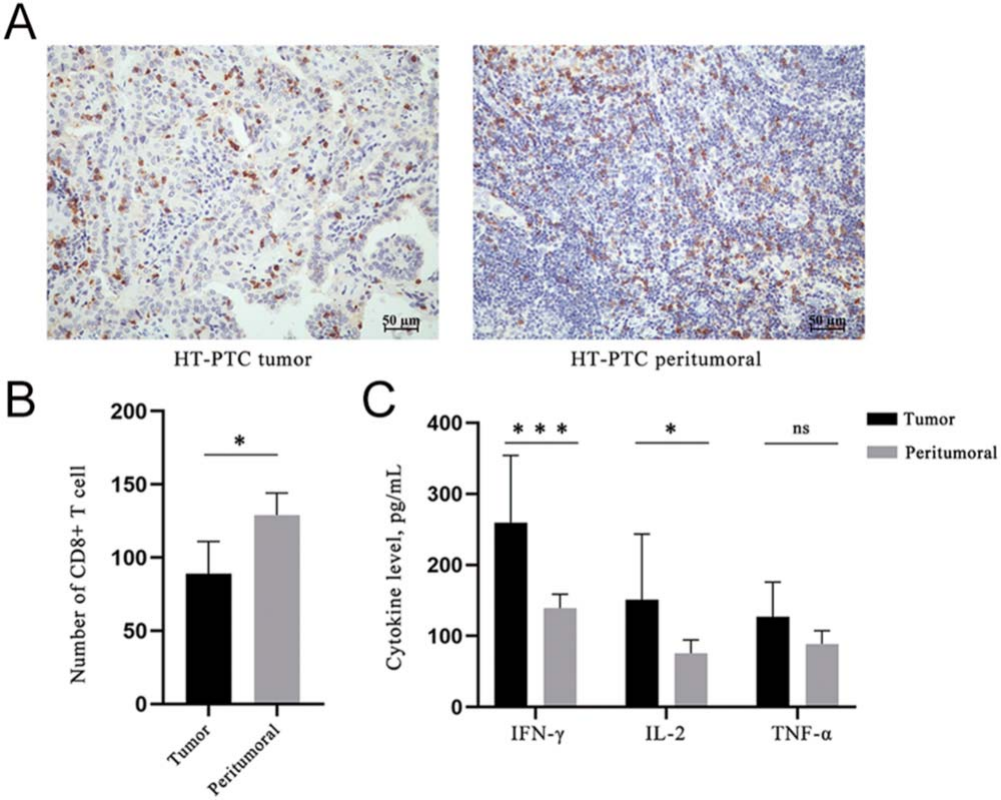
Number of CD8+ T cells and cytokine levels in tumor and peritumoral tissues from HT-PTC patients (*n* = 19). (A) Representative IHC images. (B) Quantification of CD8+ T cell numbers. (C) IFN-γ, IL-2, and TNF-α levels. *P < 0.05, ***P < 0.001; ns,: not significant.

In the HT-PTC group, the infiltration of CD8+ T cells was significantly higher in peritumoral tissues compared to tumor tissues (*P* < 0.05), showing a diffuse distribution pattern (Fig. 3A and B). Cytokine analysis revealed that IFN-γ, IL-2, and TNF-α levels were elevated in tumor tissues relative to peritumoral tissues. Among these, IFN-γ and IL-2 levels exhibited statistically significant differences, with IFN-γ showing the most pronounced increase (*P* < 0.001), whereas changes in TNF-α levels were not statistically significant (Fig. 3C).

### Isolation and functional assessment of primary CD8+ T cells

Primary CD8+ T cells were successfully isolated from HT-PTC tumor tissues, peritumoral tissues, peripheral blood of HT-PTC patients, and peripheral blood of healthy individuals using a negative selection kit. The cell purity was satisfactory, exceeding 70% in all samples (tumor tissues: 70.15%, peritumoral tissues: 71.50%, HT PTC peripheral blood: 77.83%, and healthy peripheral blood: 80.38%), with a viable cell proportion of approximately 92.6 ± 2.1%.

**Figure 4:**
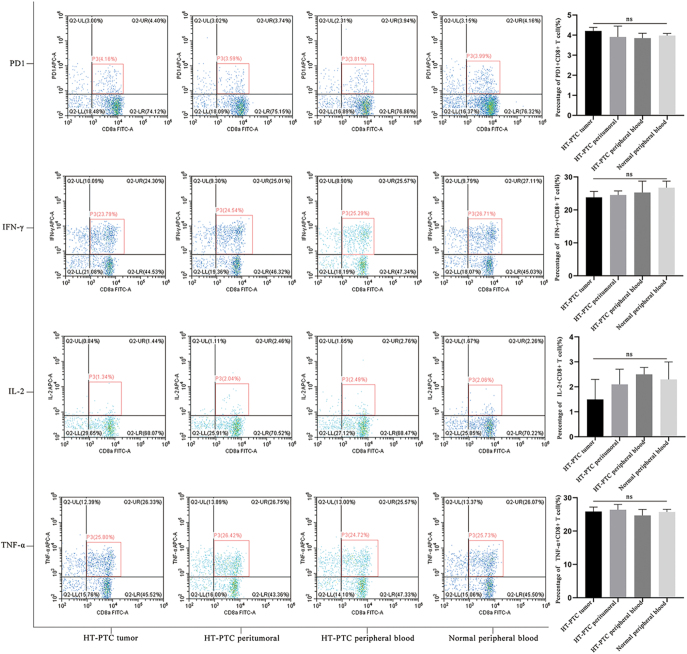
Expression of PD1 on primary CD8+ T cells from different sources and intracellular IFN-γ, IL-2, and TNF-α levels after stimulation. HT-PTC: *n* = 6, non-HT-PTC: *n* = 11, and healthy controls: *n* = 6; ns, not significant.

Figure 4 illustrates the PD1 expression levels on the surface of CD8+ T cells from different sources and their cytokine secretion capacity after stimulation. PD1 expression on HT-PTC tumor-derived CD8+ T cells was slightly elevated compared to other groups; however, the differences were not statistically significant (*P* > 0.05). Pairwise comparisons also revealed no significant differences in PD1 expression among the four groups (*P* > 0.05).

After 6 h of stimulation with PMA, ionomycin, and BFA, cytokine secretion levels of IFN-γ, IL-2, and TNF-α from HT-PTC tumor-derived CD8+ T cells were slightly lower compared to peripheral blood CD8+ T cells, but the differences were not statistically significant (*P* > 0.05). Pairwise comparisons showed no significant differences in the secretion of these cytokines among the four groups (*P* > 0.05). Additionally, the ability of CD8+ T cells to secrete IL-2 was lower compared to their secretion of IFN-γ and TNF-α.

**Figure 5:**
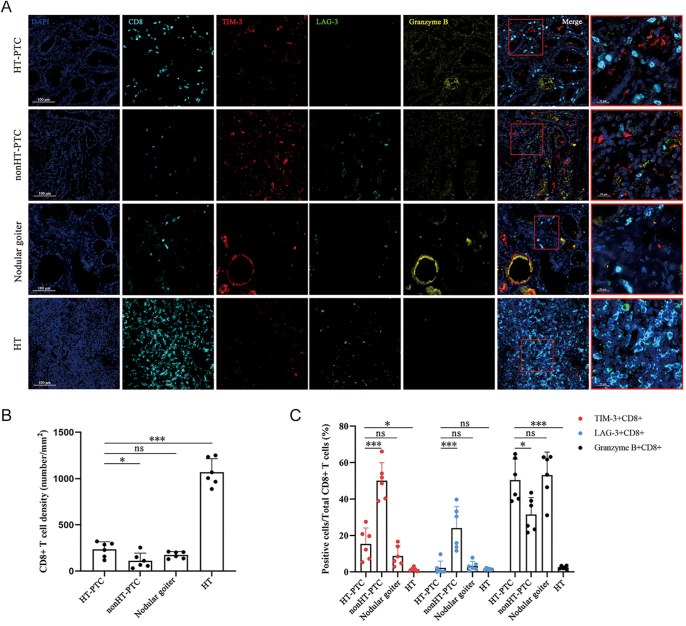
mIF profiling of CD8+ T cells in HT-PTC, non-HT-PTC, nodular goiter, and simple HT (*n* = 6 per group). CD8A (cyan), TIM-3 (red), LAG-3 (green), and Granzyme B (yellow). (A) Representative images. (B) Quantification of CD8+ T cell densities. (C) Proportion of TIM-3+CD8+, LAG-3+CD8+, and Granzyme B+CD8+ T cells among total CD8+ T cells. *P < 0.05, ***P < 0.001; ns, not significant.

Figure 5A presents representative multiplex immunofluorescence images of TIM-3, LAG-3, and Granzyme B expression on CD8+ T cells in HT-PTC, non-HT-PTC, nodular goiter, and simple HT. CD8+ T cell density in HT-PTC was significantly higher than in non-HT-PTC, lower than in simple HT, and showed no significant difference from nodular goiter (Fig. 5B). The proportion of TIM-3+CD8+ T cells in HT-PTC was significantly lower than in non-HT-PTC but higher than in simple HT (*P* = 0.010), with no significant difference compared to nodular goiter. In contrast, the proportion of LAG-3+CD8+ T cells was significantly reduced in HT-PTC compared with non-HT-PTC. Moreover, the proportion of Granzyme B+CD8+ T cells was significantly higher in HT-PTC than in non-HT-PTC (*P* = 0.011) and simple HT (Fig. 5C).

### Inhibitory effects of CD8+ T cells on TPC-1 cells at different coculture durations

**Figure 6:**
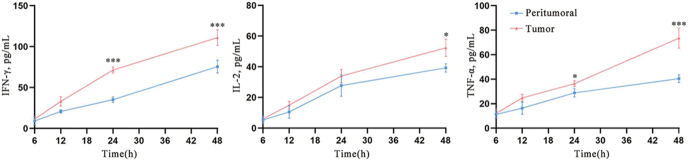
Cytokine levels (IFN-γ, IL-2, and TNF-α) in the supernatant at different coculture durations (24 and 48 h) using an effector-to-target ratio of 5:1. CD8+ T cells were derived from HT-PTC tumor tissues and peritumoral tissues (*n* = 6). *P < 0.05, ***P < 0.001.

Using an effector-to-target ratio of 5:1, the inhibitory effects of CD8+ T cells on TPC-1 cells and the secretion of cytokines in the supernatant were assessed at different coculture durations (Table 2 and Fig. 6). The inhibitory effects of HT-PTC tumor-derived CD8+ T cells on TPC-1 cells increased with extended coculture duration. At both 24 and 48 h, CD8+ T cells from HT-PTC tumor tissues exhibited significantly stronger inhibitory effects on TPC-1 cells compared to CD8+ T cells from peritumoral tissues (*P* < 0.05).

**Table 2:** Inhibition rate (%) of TPC-1 growth at different coculture times. Statistically significant values (*P<*0.05) are presented in bold.

	TPC-1 cell (E:T(5:1)) at different coculture times			
	6 h	12 h	24 h	48 h
CD8+ T cell origin				
HT-PTC tumor tissue	0.45 ± 0.20	8.71 ± 0.51	47.22 ± 3.56	88.01 ± 1.78
HT-PTC peritumoral tissue	0.37 ± 0.44	6.99 ± 0.24	24.30 ± 2.19	36.53 ± 2.20
Statistic	0.40	1.00	8.63	20.59
Effect size*	0.066	0.41	1.70	2.01
95% CI^†^	−0.0038∼0.12	1.25–2.03	20.86–24.05	50.40–54.74
*P*	0.88	0.105	**0.026**	**<0.001**

* Cohen’s d.

^†^ 95% confidence interval for the mean difference.

Regarding cytokine secretion, the levels of IFN-γ secreted by HT-PTC tumor-derived CD8+ T cells were significantly higher than those secreted by peritumoral tissue-derived CD8+ T cells at 24 and 48 h (*P* < 0.001). Additionally, TNF-α secretion was also significantly increased. In contrast, IL-2 differences reached statistical significance only after 48 h of coculture (*P* < 0.05).

### Assessment of the effects of primary CD8+ T cells on TPC-1 cell proliferation and migration

The effects of CD8+ T cells on the proliferation and migration of TPC-1 cells were evaluated after coculture at a 5:1 effector-to-target ratio for 48 h. The CCK8 assay demonstrated that the viability of TPC-1 cells cocultured with tumor-derived CD8+ T cells was significantly reduced on days 5, 6, and 7 compared to the peritumoral group and the blank control group (*P* < 0.05).

**Figure 7:**
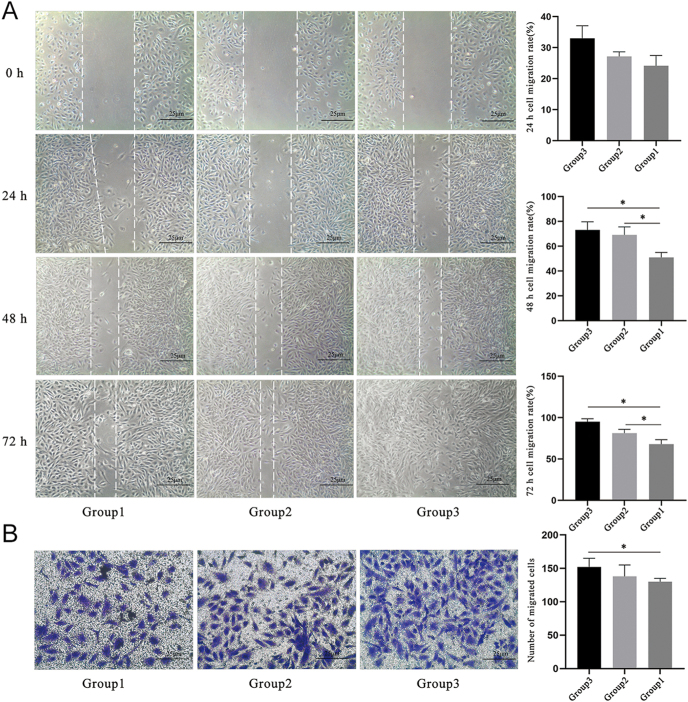
Wound healing and Transwell migration assays (*n* = 6). (A) Wound healing assay. (B) Transwell migration assay. Group 1: TPC-1 cells cocultured with CD8+ T cells derived from HT-PTC tumor tissues (48 h). Group 2: TPC-1 cells cocultured with CD8+ T cells derived from HT-PTC peritumoral tissues (48 h). Group 3: blank TPC-1 cell control. *P < 0.05.

Figure 7 presents the results of the wound healing assay and Transwell assay. Compared to the blank TPC-1 control, TPC-1 cells cocultured with tumor-derived CD8+ T cells exhibited significantly reduced migration rates at 48 and 72 h, as well as significantly fewer cells migrating to the lower chamber (*P* < 0.05). At 24 h, a declining trend in migration rate was observed, but the difference did not reach statistical significance.

When compared with the peritumoral-derived CD8+ T cell group, TPC-1 cells cocultured with tumor-derived CD8+ T cells showed significantly lower migration rates at 48 and 72 h (*P* < 0.05), whereas the number of cells migrating to the lower chamber showed a nonsignificant decreasing trend. In contrast, the blank control group showed higher migration than both coculture groups, although the difference compared with the peritumoral-derived CD8+ T cell group did not reach statistical significance.

### Differential gene expression and functional enrichment analysis between HT-PTC and non-HT-PTC

**Figure 8:**
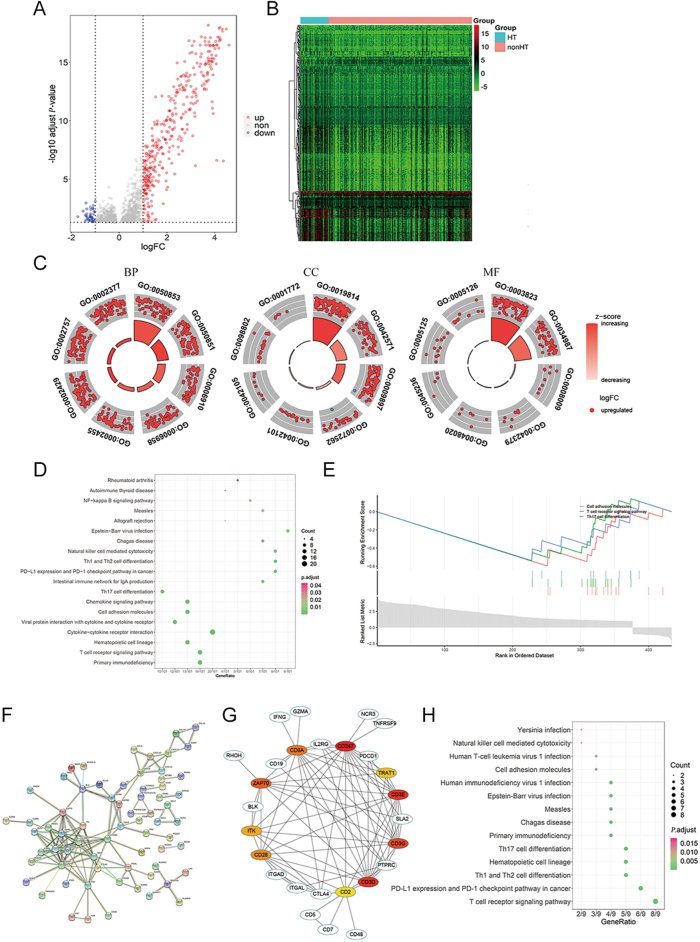
Differential gene expression and pathway enrichment analysis between HT-PTC and non-HT-PTC. (A and B) Volcano plot and heatmap of DEGs. (C and D) GO and KEGG enrichment analyses based on ORA. (E) KEGG pathway enrichment based on GSEA. (F and G) PPI network of DEGs and identification of top hub genes. (H) KEGG pathway enrichment analysis of hub genes.

To explore the molecular features associated with HT-PTC, we analyzed transcriptomic differences between HT-PTC and non-HT-PTC using TCGA data. A total of 410 DEGs were identified, including 369 upregulated and 41 downregulated genes (Fig. 8A and B).

Over-representation analysis (ORA) revealed that these DEGs were significantly enriched in 291 biological process (BP) terms (Fig. 8C, Supplementary Table S3), mainly involving the B cell receptor signaling pathway, antigen receptor-mediated signaling, phagocytosis, activation of immune response via cell surface receptor signaling, and signal transduction activation (*P* < 0.001). In terms of cellular component (CC), the DEGs were primarily associated with the immunoglobulin complex, external side of plasma membrane, blood microparticles, and α-β T cell receptor complex (*P* < 0.001). For molecular function (MF), the DEGs were mainly enriched in antigen binding, immunoglobulin receptor binding, and chemokine receptor activity (*P* < 0.001).

KEGG pathway analysis indicated that the DEGs were enriched in 19 immune-related pathways, including cytokine–cytokine receptor interaction, T cell receptor signaling pathway, and chemokine signaling pathway (Fig. 8D, Supplementary Table S4). GSEA results (Supplementary Table S5) were largely consistent with the ORA findings. Upregulated genes were predominantly associated with extracellular immune-related proteins involved in adaptive immune processes and were significantly enriched in pathways such as cell adhesion molecules, T cell receptor signaling, and Th17 cell differentiation (Fig. 8E, Supplementary Table S6).

To further investigate protein-level associations, a PPI network was constructed using the STRING database, comprising 187 nodes and 124 edges (Fig. 8F). Based on the MCC algorithm in the CytoHubba plugin, the top 10 hub genes were identified: CD247, CD3E, CD3D, CD3G, ZAP70, CD8A, CD28, ITK, TRAT1, and CD2 (Fig. 8G) – all of which are closely related to T cell receptor signaling and activation. KEGG enrichment of these hub genes (Fig. 8H) was consistent with their predominant involvement in T cell-related immune pathways.

## Discussion

Multiple clinical studies have shown that PTC patients with coexisting HT often display less aggressive features, including lower lymph node metastasis and better prognosis. These observations are further supported by experimental evidence from a BRAF-driven mouse model, in which preexisting thyroiditis was associated with lower PTC incidence, prolonged survival, less aggressive histopathological features, and increased intratumoral CD8+ T cell infiltration (15). In our analysis of TCGA data, HT-PTC similarly exhibited a more indolent clinicopathological profile, with a higher proportion of N0 disease, less extrathyroidal extension, and earlier AJCC stage. These findings are consistent with prior clinical observations. Against this clinical background, we investigated whether CD8+ T cell infiltration and functional status may be associated with this less aggressive behavior. Transcriptomic analysis revealed significantly higher CD8+ T cell infiltration in the HT-PTC, consistent with the dense lymphocytic environment characteristic of HT (16). Given that HT is marked by sustained autoimmune activation involving autoreactive T cells and B cell-mediated antibody responses (17, 18), such immune alterations may contribute to shaping a tumor microenvironment distinct from that of non-HT-PTC.

We further validated the infiltration and characteristics of CD8+ T cells in HT-PTC. CD8A, encoding the CD8 antigen, serves as a key marker for cytotoxic CD8+ T cells (19). CD8A mRNA and protein expression were significantly elevated in the HT-PTC group and the PTC patients without LNM. Flow cytometry confirmed higher CD8+ T cell infiltration in the tumor and peritumoral tissues of HT-PTC. Prior studies have linked elevated CD8+ T cell levels with a favorable prognosis in thyroid cancer (20). However, Banerjee *et al.* (21) noted that despite high lymphocyte infiltration, PD1/PD-L1 expression was also elevated in HT-PTC, potentially impairing T cell function. To further characterize the functional state of these cells, we evaluated CD8+ T cell functionality and cytotoxicity against PTC cells.

In HT-PTC, CD8+ T cells infiltrated peritumoral tissues more than tumor tissues, yet IFN-γ, IL-2, and TNF-α levels were significantly higher in tumor tissues, especially IFN-γ and IL-2 – suggesting an activated immune milieu. In coculture assays, IFN-γ and TNF-α increased significantly over time, while IL-2 rose notably after 48 h, consistent with previous findings (22), implying that IL-2 in tumors may originate from other immune cells. Hu *et al.* (23) showed that IL-2 enhances MHC-I expression on PTC cells under HT conditions, aiding CD8+ T cell recognition. IFN-γ suppresses CXCL8 and induces CXCL10, reducing tumor migration (24). These cytokines may be associated with the inhibition of tumor growth in HT-PTC. However, TNF-α can promote CCR6 expression on PTC cells, potentially enhancing invasion and metastasis (25). Thus, despite an overall antitumor milieu, elevated TNF-α reflects the complex immune landscape in HT-PTC.

Flow cytometry showed that tumor-derived CD8+ T cells from HT-PTC had slightly upregulated PD-1 and modestly reduced IFN-γ, IL-2, and TNF-α secretion compared to those in peripheral blood, though differences were not statistically significant. In contrast, CD8+ T cells in peritumoral tissues secreted slightly more cytokines and expressed less PD1, suggesting the presence of some degree of local functional suppression within the tumor core microenvironment. While TIM-3+CD8+ T cells were increased in HT-PTC vs simple HT, their proportion remained lower than in non-HT-PTC; LAG-3+CD8+ T cells were also reduced. Conversely, Granzyme B+CD8+ T cells were markedly elevated, suggesting preserved cytotoxic capacity. Taken together, these findings indicate that CD8+ T cells in HT-PTC display limited exhaustion-associated features rather than overt terminal exhaustion. Despite mild PD1 upregulation, their cytokine-secreting ability shows only modest impairment. Notably, reliance on PD-1 expression in isolation is inadequate to conclude true exhaustion. Instead, the lower expression of multiple inhibitory receptors (particularly TIM-3 and LAG-3), combined with significantly elevated Granzyme B, supports the interpretation of relatively preserved functionality. Previous studies indicate that T cells can retain partial activity despite PD1 or CTLA4 expression (26), and that CD8+ T cell exhaustion correlates with deficient CD4+ T cell (27, 28). Given the robust CD4+ T cell infiltration characteristic of HT, this may contribute to supporting CD8+ T cell function in the HT-PTC, although further experimental validation is required.

In coculture with TPC-1 cells, CD8+ T cells from HT-PTC exhibited strong cytotoxicity, progressively inhibiting tumor proliferation and migration, with tumor-derived CD8+ T cells showing the strongest effect. CD8+ T cells from peritumoral tissues were slightly less potent but still significantly suppressed tumor growth. These results support that HT may be associated with reduced PTC aggressiveness through enhanced CD8+ T cell infiltration and function. This immune-preserved phenotype may partly contribute to the lower lymph node metastasis rate and earlier clinical stage observed in HT-PTC patients, highlighting a potential immune basis for their distinct clinical behavior. However, the suppressive effect of HT on tumor growth requires further *in vivo* validation. We are currently developing an orthotopic thyroid carcinoma model in immunocompetent mice, with HT-like autoimmunity induced via exogenous thyroglobulin, to investigate HT-driven immune modulation under physiological conditions.

To explore molecular pathways potentially associated with CD8+ T cell regulation in HT-PTC, we identified 410 DEGs between HT-PTC and non-HT-PTC. ORA and GSEA showed enrichment in immune pathways such as TCR signaling, cytokine–cytokine receptor interaction, and chemokine signaling. PPI analysis identified key hub genes (CD3E, CD3G, ZAP70, and ITK) closely related to T cell activation (29), which were consistent with the involvement of TCR–NF-κB signaling. Recent single-cell transcriptomics in HT have suggested that CD8+ T cell–macrophage interaction via the CD70–CD27 axis may activate TCR–NF-κB cascades (30). Building on these insights, we plan to incorporate single-cell RNA sequencing and spatial transcriptomics in future studies to dissect CD8+ T cell heterogeneity across tumor compartments and identify key signaling pathways that may regulate CD8+ T cell activity in HT-PTC.

This study has several limitations. First, the analysis was restricted to classical PTC and its follicular variant, limiting the generalizability of our findings to more aggressive histological subtypes of PTC. Second, while we evaluated checkpoint receptor expression and cytokine production, these parameters alone do not fully characterize bona fide T cell exhaustion. A more comprehensive understanding of the underlying mechanisms would require additional approaches. Furthermore, the functional experiments in this study were conducted exclusively *in vitro*. Future investigations incorporating spatial transcriptomics, single-cell RNA sequencing, and relevant *in vivo* models are, therefore, needed to better elucidate the immunological microenvironment and T cell dynamics in HT-PTC.

## Conclusion

In HT-PTC, tumor-derived CD8+ T cells exhibited relatively preserved cytokine secretion and cytotoxic activity and were associated with reduced tumor cell proliferation and migration *in vitro*. These findings indicate a potential immune basis for the less aggressive clinical phenotype of HT-PTC and provide preliminary biological support for future immune-informed risk stratification.

## Supplementary materials























## Declaration of interest

The authors declare that there is no conflict of interest that could be perceived as prejudicing the impartiality of the research reported.

## Funding

This work was supported by the National Natural Science Foundation of China (Nos. 82202183 and 82071952), the IIT Clinical Research Fund of the Second Affiliated Hospital of Xi’an Jiaotong University (2025IIT-T02) , and the Youth Project of the Hospital Fund of the Second Affiliated Hospital of Xi'an Jiaotong University (YJ (QN) 202525).

## Author contribution statement

WLR, WJ, and JJ conceptualized and designed the study and drafted the initial manuscript. WLR, ZL, and CJW contributed to manuscript writing. ZTY and WD were responsible for data analysis. SL and SWX designed and optimized the experimental procedures. LM provided overall guidance on manuscript preparation. ZQ supervised the experimental work and critically revised the manuscript. All authors reviewed and approved the final version of the article and agreed to its submission.

## References

[1] Chen DW & Haymart MR. Unravelling the rise in thyroid cancer incidence and addressing overdiagnosis. Nat Rev Endocrinol 2026 22 10–20. (10.1038/s41574-025-01168-y) 40903496 PMC12814954

[2] Bray F, Laversanne M, Sung H, et al. Global cancer statistics 2022: GLOBOCAN estimates of incidence and mortality worldwide for 36 cancers in 185 countries. CA Cancer J Clin 2024 74 229–263. (10.3322/caac.21834) 38572751

[3] Mao J, Zhang Q, Zhang H, et al. Risk factors for lymph node metastasis in papillary thyroid carcinoma: a systematic review and meta-analysis. Front Endocrinol 2020 11 11265. (10.3389/fendo.2020.00265) PMC7242632 32477264

[4] Elisei R & Pinchera A. Advances in the follow-up of differentiated or medullary thyroid cancer. Nat Rev Endocrinol 2012 8 466–475. (10.1038/nrendo.2012.38) 22473335

[5] Ugolini C, Basolo F, Proietti A, et al. Lymphocyte and immature dendritic cell infiltrates in differentiated, poorly differentiated, and undifferentiated thyroid carcinoma. Thyroid 2007 17 389–393. (10.1089/thy.2006.0306) 17542668

[6] Xu J, Ding K, Mu L, et al. Hashimoto’s thyroiditis: Aa“double-edged sword” in thyroid carcinoma. Front Endocrinol 2022 13 13801925. (10.3389/fendo.2022.801925) PMC8907134 35282434

[7] Wang Y, Zheng J, Hu X, et al. A retrospective study of papillary thyroid carcinoma: Hashimoto’s thyroiditis as a protective biomarker for lymph node metastasis. Eur J Surg Oncol 2023 49 560–567. (10.1016/j.ejso.2022.11.014) 36404253

[8] Zeng B, Min Y, Feng Y, et al. Hashimoto’s thyroiditis is associated with central lymph node metastasis in classical papillary thyroid cancer: analysis from a high-volume single-center experience. Front Endocrinol 2022 13 13868606. (10.3389/fendo.2022.868606) PMC9185947 35692401

[9] Zhang QY, Ye XP, Zhou Z, et al. Lymphocyte infiltration and thyrocyte destruction are driven by stromal and immune cell components in Hashimoto’s thyroiditis. Nat Commun 2022 13 775. (10.1038/s41467-022-28120-2) 35140214 PMC8828859

[10] Ehlers M, Kuebart A, Hautzel H, et al. Epitope-specific antitumor immunity suppresses tumor spread in papillary thyroid cancer. J Clin Endocrinol Metab 2017 102 2154–2161. (10.1210/jc.2016-2469) 27860539

[11] Baessler A & Vignali DAA. T cell exhaustion. Annu Rev Immunol 2024 42 179–206. (10.1146/annurev-immunol-090222-110914) 38166256

[12] Severson JJ, Serracino HS, Mateescu V, et al. PD-1+Tim-3+ CD8+ T lymphocytes display varied degrees of functional exhaustion in patients with regionally metastatic differentiated thyroid cancer. Cancer Immunol Res 2015 3 620–630. (10.1158/2326-6066.cir-14-0201) 25701326 PMC4457654

[13] Newman AM, Liu CL, Green MR, et al. Robust enumeration of cell subsets from tissue expression profiles. Nat Methods 2015 12 453–457. (10.1038/nmeth.3337) 25822800 PMC4739640

[14] Ringel MD, Sosa JA, Baloch Z, et al. 2025 American Thyroid Association Management Guidelines for adult patients with differentiated thyroid cancer. Thyroid 2025 35 841–985. (10.1177/10507256251363120) 40844370 PMC13090833

[15] Pani F, Yasuda Y, Di Dalmazi G, et al. Pre-existing thyroiditis ameliorates papillary thyroid cancer: insights from a new mouse model. Endocrinology 2021 162 bqab144. (10.1210/endocr/bqab144) 34331442 PMC8389179

[16] Ralli M, Angeletti D, Fiore M, et al. Hashimoto’s thyroiditis: an update on pathogenic mechanisms, diagnostic protocols, therapeutic strategies, and potential malignant transformation. Autoimmun Rev 2020 19 102649. (10.1016/j.autrev.2020.102649) 32805423

[17] McLachlan SM & Rapoport B. Breaking tolerance to thyroid antigens: changing concepts in thyroid autoimmunity. Endocr Rev 2014 35 59–105. (10.1210/er.2013-1055) 24091783 PMC3895862

[18] Smith TJ & Hegedüs L. Graves’ disease. N Engl J Med 2016 375 1552–1565. (10.1056/nejmra1510030) 27797318

[19] Zheng Z, Guo Y, Huang X, et al. CD8A as a prognostic and immunotherapy predictive biomarker can be evaluated by MRI radiomics features in bladder cancer. Cancers 2022 14 4866. (10.3390/cancers14194866) 36230788 PMC9564077

[20] Yang Z, Wei X, Pan Y, et al. A new risk factor indicator for papillary thyroid cancer based on immune infiltration. Cell Death Dis 2021 12 51. (10.1038/s41419-020-03294-z) 33414407 PMC7791058

[21] Banerjee S, Nahar U, Dahiya D, et al. Role of cytotoxic T cells and PD-1 immune checkpoint pathway in papillary thyroid carcinoma. Front Endocrinol 2022 13 13931647. (10.3389/fendo.2022.931647) PMC9742369 36518249

[22] Okda TM, Atwa GMK, Eldehn AF, et al. A novel role of Galectin-3 and thyroglobulin in prognosis and differentiation of different stages of thyroid cancer and elucidation of the potential contribution of Bcl-2, IL-8 and TNF-α. Biomedicines 2022 10 352. (10.3390/biomedicines10020352) 35203561 PMC8962323

[23] Hu JQ, Lei BW, Wen D, et al. IL-2 enhanced MHC class I expression in papillary thyroid cancer with Hashimoto’s thyroiditis overcomes immune escape in vitro. J Cancer 2020 11 4250–4260. (10.7150/jca.38330) 32368308 PMC7196247

[24] Rotondi M, Coperchini F, Awwad O, et al. Effect of Interferon-γ on the basal and the TNFα-stimulated secretion of CXCL8 in thyroid cancer cell lines bearing either the RET/PTC rearrangement or the BRAF V600e mutation. Mediators Inflamm 2016 2016 20168512417. (10.1155/2016/8512417) PMC4983361 27555670

[25] Coperchini F, Pignatti P, Carbone A, et al. TNF-α increases the membrane expression of the chemokine receptor CCR6 in thyroid tumor cells, but not in normal thyrocytes: potential role in the metastatic spread of thyroid cancer. Tumour Biol 2016 37 5569–5575. (10.1007/s13277-015-4418-7) 26577851

[26] Tallón de Lara P, Castañón H, Vermeer M, et al. CD39(+)PD-1(+)CD8(+) T cells mediate metastatic dormancy in breast cancer. Nat Commun 2021 12 769. (10.1038/s41467-021-21045-2) 33536445 PMC7859213

[27] Cui C, Wang J, Fagerberg E, et al. Neoantigen-driven B cell and CD4 T follicular helper cell collaboration promotes anti-tumor CD8 T cell responses. Cell 2021 184 6101–6118.e13. (10.1016/j.cell.2021.11.007) 34852236 PMC8671355

[28] Magen A, Hamon P, Fiaschi N, et al. Intratumoral dendritic cell-CD4(+) T helper cell niches enable CD8(+) T cell differentiation following PD-1 blockade in hepatocellular carcinoma. Nat Med 2023 29 1389–1399. (10.1038/s41591-023-02345-0) 37322116 PMC11027932

[29] Deng S, Zhang Y, Wang H, et al. ITPRIPL1 binds CD3ε to impede T cell activation and enable tumor immune evasion. Cell 2024 187 2305–2323.e33. (10.1016/j.cell.2024.03.019) 38614099

[30] Zheng H, Xu J, Chu Y, et al. A global regulatory network for dysregulated gene expression and abnormal metabolic signaling in immune cells in the microenvironment of Graves’ disease and Hashimoto’s thyroiditis. Front Immunol 2022 13 13879824. (10.3389/fimmu.2022.879824) PMC9204353 35720300

