# Supplementary figures and images for: CD8+ T cells in Hashimoto’s thyroiditis-associated papillary thyroid carcinoma
Source: Eur Thyroid J. 2026 Jun 9;15(3):ETJ250365. doi: 10.1530/ETJ-25-0365 (PMC13261502; doi:10.1530/ETJ-25-0365)

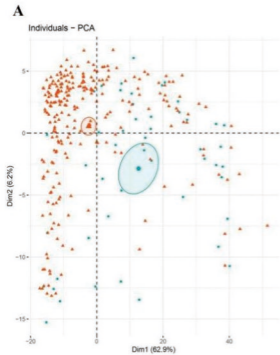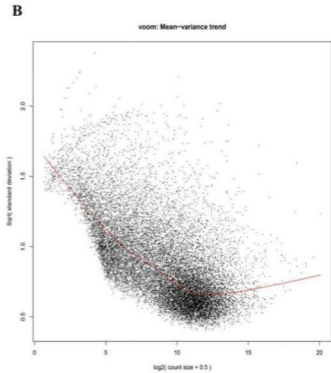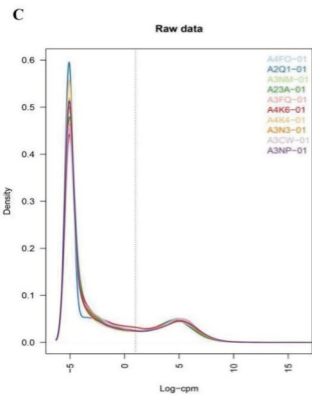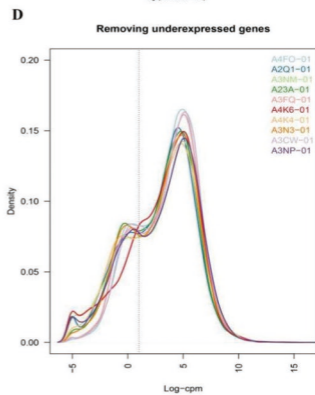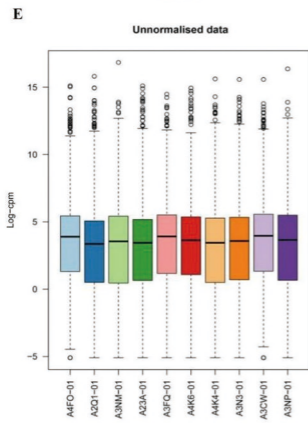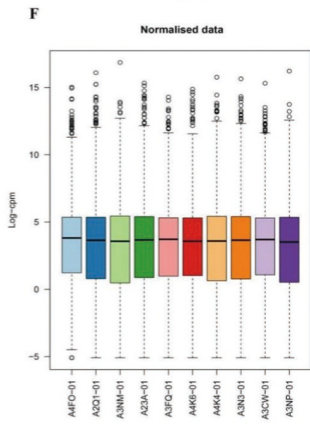

Supplement: Supplementary file 1 [file supplementary_figure_1.pdf]

A

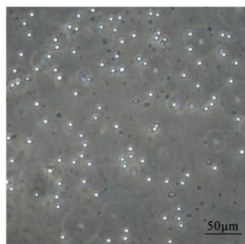

B

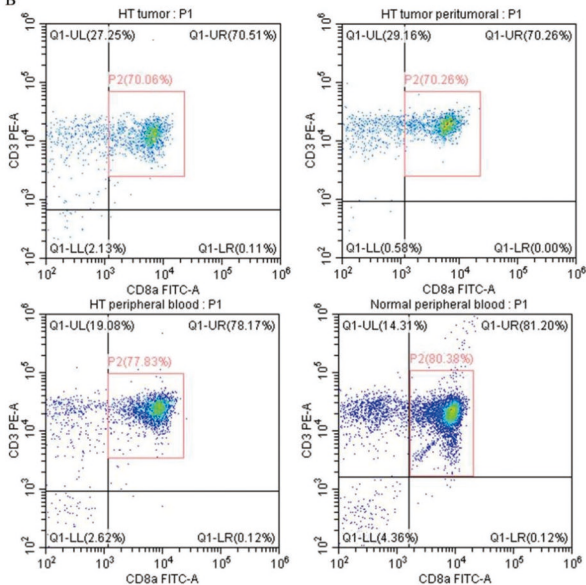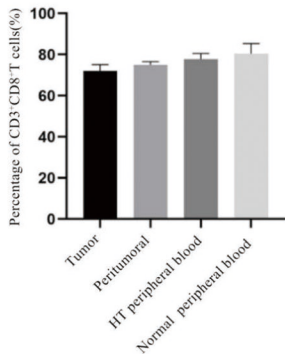

Supplement: Supplementary file 2 [file supplementary_figure_2.pdf]

SSC-A :: SSC-A

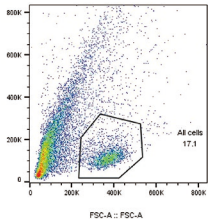

FSC-H :: FSC-H

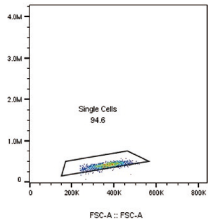

SSC-A :: SSC-A

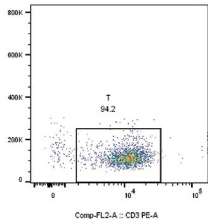IFN1.fcs  
Ungated  
14089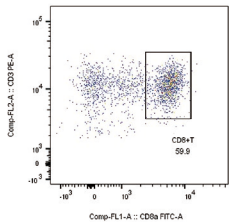IFN1.fcs  
All cells  
2405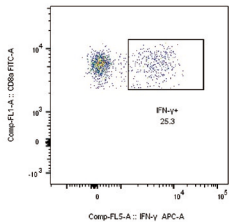IFN1.fcs  
T  
2142IFN1.fcs  
CD8+T  
1283

Supplement: Supplementary file 3 [file supplementary_figure_3.pdf]
