# Supplementary material for: CD8+ T cells in Hashimoto’s thyroiditis-associated papillary thyroid carcinoma
Source: Eur Thyroid J. 2026 Jun 9;15(3):ETJ250365. doi: 10.1530/ETJ-25-0365 (PMC13261502; doi:10.1530/ETJ-25-0365)
Supplement: Supplementary file 4 [file supplementary_table_1.pdf]

Table S1 Clinical characteristics of PTC from our center

**Characteristics**	HT-PTC (*n*=19)	nonHT-PTC (*n*=52)	Statistics	*P*
**Age, years**	45.00±12.25	44.1±10.03	0.310	0.758 [a]
**Sex**			1.647	0.199 [b]
Male	4 (21.1)	19 (37.3)		
Female	15 (78.9)	32 (62.7)		
**TPO-Ab, IU/mL**	28.20 (15.00, 91.90)	9.06 (9.00, 12.10)	5.130	**<0.001** [c]
**Tg-Ab, IU/mL**	212.00 (70.50, 325.00)	13.16 (10.90, 16.00)	6.283	**<0.001** [c]
**TSH, μIU/mL**	2.35±1.17	2.54±1.40	0.589	0.558 [a]
**FT3，pmol/L**	4.80±0.56	5.20±2.22	0.761	0.449 [a]
**FT4，pmol/L**	16.32±2.35	16.32±3.29	0.007	0.994 [a]
**Maximum tumor diameter, mm**	9.90 (8.70, 14.60)	13.05 (9.53, 17.93)	1.442	0.149 [c]
**Type of surgery**			0.108	0.742 [b]
Total thyroidectomy	2 (10.5)	7 (13.5)		
Lobectomy	17 (89.5)	45 (86.5)		
**Tumor location**			3.860	0.277 [b]
Left lobe	4 (21.1)	22 (42.3)		

Right lobe	11 (57.9)	21 (40.4)		
Isthmus	2 (10.5)	2 (3.8)		
Bilateral	2 (10.5)	7 (13.5)		
**Histological type**			0.125	0.723 [b]
Classical	18 (94.7)	48 (92.3)		
Follicular	1 (5.3)	4 (7.7)		
**Focus type**			0.073	0.787 [b]
Multifocal	7 (36.8)	21 (40.4)		
Unifocal	12 (63.2)	31 (59.6)		
**Extrathyroidal extension**			0.617	0.432 [b]
Yes	1 (5.3)	6 (11.5)		
No	18 (94.7)	46 (88.5)		
**N stage**			7.672	**0.006** [b]
0	10 (52.6)	10 (19.2)		
1	9 (47.2)	42 (80.8)		
Risk of recurrence			1.486	0.791 [d]
Low	14 (73.7)	34 (65.4)		
Low-Intermediate	3 (15.8)	6 (11.5)		
Intermediate-High	1 (5.3)	8 (15.4)		
High	1 (5.3)	4 (7.7)		

a, independent samples t-test; b, χ^2 test; c, Mann-Whitney U test; d, Fisher's exact test.
