# Supplementary material for: CD8+ T cells in Hashimoto’s thyroiditis-associated papillary thyroid carcinoma
Source: Eur Thyroid J. 2026 Jun 9;15(3):ETJ250365. doi: 10.1530/ETJ-25-0365 (PMC13261502; doi:10.1530/ETJ-25-0365)
Supplement: Supplementary file 5 [file supplementary_table_2.pdf]

Table S2 Clinical characteristics of the PTC subset included in functional CD8+ T cell assays

**Characteristics**	HT-PTC (n= 6)	nonHT-PTC (n=11)
**Age, years**	44.83±12.04	40.18±8.96
**Sex**		
Male	3 (50.0)	6 (54.5)
Female	3 (50.0)	5 (45.5)
**TPO-Ab, IU/mL**	181.65 (44.40, 595.68)	11.10 (9.00, 16.50)
**Tg-Ab, IU/mL**	321.25 (173.84, 1793.5)	13.16 (10.90, 16.00)
**TSH, µIU/mL**	2.90±1.35	2.31±1.15
**Maximum tumor diameter, mm**	15.30 (10.60, 20.38)	15.70 (13.80, 21.60)
**Type of surgery**		
Total thyroidectomy	1 (16.7)	0 (0)
Lobectomy	5 (83.3)	11 (100)
**Tumor location**		
Left lobe	1 (16.7)	3 (27.3)
Right lobe	4 (66.6)	7 (63.6)
Isthmus	0 (0)	1 (9.1)

Bilateral	1 (16.7)	0 (0)
**Histological type**		
Classical	6 (100)	11(100)
**Focus type**		
Multifocal	1(16.7)	2 (18.2)
Unifocal	5 (83.3)	9 (81.8)
**Extrathyroidal extension**		
Yes	0 (0)	1 (9.1)
No	6 (100)	10 (90.9)
**N stage**		
0	2 (33.6)	4 (36.4)
1	4 (66.4)	7 (63.6)

This nested subcohort was selected based on tumor size (≥1 cm) to ensure adequate tissue for ex vivo assays; therefore, baseline comparisons were descriptive.
