# Supplementary material for: CD8+ T cells in Hashimoto’s thyroiditis-associated papillary thyroid carcinoma
Source: Eur Thyroid J. 2026 Jun 9;15(3):ETJ250365. doi: 10.1530/ETJ-25-0365 (PMC13261502; doi:10.1530/ETJ-25-0365)
Supplement: Supplementary file 10 [file supplementary_table_7.pdf]

Table S7 Recurrence risk stratification of PTC from our center by histological type

**Risk of recurrence**	HT-PTC (*n*=19)		nonHT-PTC (*n*=52)	
	Classical	Follicular	Classical	Follicular
Low	13 (72.2)	1 (100.0)	31 (64.6)	3 (75.0)
Low-Intermediate	3 (16.7)	0 (0)	6 (12.5)	0 (0)
Intermediate-High	1 (5.6)	0 (0)	7 (14.6)	1 (25.0)
High	1 (5.6)	0 (0)	4 (8.3)	0 (0)
