# Supplementary material for: CD8+ T cells in Hashimoto’s thyroiditis-associated papillary thyroid carcinoma
Source: Eur Thyroid J. 2026 Jun 9;15(3):ETJ250365. doi: 10.1530/ETJ-25-0365 (PMC13261502; doi:10.1530/ETJ-25-0365)
Supplement: Supplementary file 11 [file supplementary_materials.pdf]

R Code for Bioinformatics Analysis

1 Data Processing

1.1 Total mRNA Expression Data of PTC Patients

```
# Set working directory

setwd("C:\\Users\\ Desktop\\mRNAprocessing")

library("rjson")

rm(list = ls())

options(stringsAsFactors = F)


# Process metadata file

result <- fromJSON(file = "metadata.cart.2022-04-17.json")

metadata <- data.frame(t(sapply(result, function(x){

        id <- x$associated_entities[[1]]$entity_submitter_id

        file_name <- x$file_name

        all <- cbind(id, file_name)})))

rownames(metadata) <- metadata[, 2]


# Generate mRNA matrix

dir <- './all/'

samples = list.files(dir)

sampledir <- paste0(dir, samples)

mat <- do.call(cbind, lapply(sampledir, function(x){
```

```
  rt <- data.table::fread(x, data.table = F)
  rownames(rt) <- rt[, 1]
  rt <- rt[, 4]})) # Extract the 4th column values

# Row and column name conversion
rt <-
data.table::fread('./all/00f54652-691e-4446-869a-4dd51c236f56.rna_seq.augmented_star_gene_co
unts.tsv', data.table = F)
colnames(mat) = sapply(strsplit(sampledir, '/'), '[', 3)
rownames(mat) <- rt$gene_id
mat1 <- t(mat)

same <- intersect(row.names(metadata), row.names(mat1))
data <- cbind(metadata[same,], mat1[same,])
rownames(data) <- data[, 1]
tcga_stad <- t(data)
tcga_stad <- tcga_stad[-c(1:6),]
rownames(rt) <- rt[, 1]

# Gene ID conversion
same2 <- intersect(row.names(rt), row.names(tcga_stad))
tcga <- cbind(rt[same2,], tcga_stad[same2,])
```

```
tcga <- tcga[-c(1, 4:9)]


# Save mRNA matrix file

write.csv(tcga, file="tcga.csv", row.names=F, quote = F)

write.table(tcga, file="tcga.txt", sep="\t", row.names=F, quote=F)


# Extract target matrix (HT_nonHT)

id_ht <- scan(file="id_ht.txt", what = "")

data <- read.csv("tcga.csv", check.names = F)

ht_exp <- data[, colnames(data) %in% id_ht]

write.csv(ht_exp, file="ht_exp.csv", row.names=F, quote = F)


# Format data, remove duplicates, and save

library(limma), library(edgeR)


# Import data

data <- read.csv("merge_exp.csv", check.names = F)


# Format data

data = as.matrix(data)

rownames(data) = data[, 1]

exp = data[, 2:ncol(data)]
```

```
# Convert to numerical matrix

dimnames = list(rownames(exp), colnames(exp))

data = matrix(as.numeric(as.matrix(exp)), nrow=nrow(exp), dimnames=dimnames)


# Remove duplicates and take average if multiple rows represent the same gene

data = avereps(data)


# Separate HT and non-HT matrices based on IDs

id_ht <- scan(file="id_ht.txt", what = "")

ht_clear <- data[, colnames(data) %in% id_ht]


# Save merge, HT, and non-HT matrices

write.csv(data, file="merge_clear.csv")
```

1.2 Clinical Data of PTC Patients

```
setwd("C:\\Users\\ Desktop\\mRNAprocessing")

library("XML"), library("methods")

dir = "C:\\Users\\ Desktop\\mRNAprocessing"

all_files = list.files(path = dir, pattern='*.xml$', recursive=T)


# Import and process files
```

```
cl = lapply(all_files, function(x){
        result <- xmlParse(file = file.path(dir, x))
        rootnode <- xmlRoot(result)
        xmldataframe <- xmlToDataFrame(rootnode[2])
        return(t(xmldataframe)) })
clinical <- t(do.call(cbind, cl))

# Save clinical data
write.table(clinical, file="clinical.txt", sep="\t", quote=F, row.names = F)
```

1.3 Data Normalization

```
setwd("C:\\Users\\ Desktop\\mRNAprocessing")
library(limma), library(edgeR), library("RColorBrewer"), library(factoextra)
rm(list = ls())

data <- read.csv("merge_clear.csv", row.names = 1, check.names = F)
data = as.matrix(data)

# Density plot for HT/non-HT groups (first 10 columns each)
ht <- data[, 1:10]
nonht <- data[, 50:59]
```

```
# Plot density before normalization
ht_lcmp <- cpm(ht, log=T)
nonht_lcmp <- cpm(nonht, log=T)


# Plot normalized data
design <- model.matrix(~0 + group)
dge <- DGEList(counts = data, group = group)
exp_dge <- rowSums(cpm(dge) > 1) >= 12
dge <- dge[exp_dge, , keep.lib.sizes = FALSE]


# Boxplots before and after normalization
a1 <- data1[, 46:55]
lcpm_a1 <- cpm(a1, log=TRUE)
boxplot(lcpm_a1, main="Unnormalized data")


a1 <- calcNormFactors(a1)
lcpm_a1 <- cpm(a1, log=TRUE)
boxplot(lcpm_a1, main="Normalized data")
```

1.4 Immune Cell Infiltration Analysis

```
setwd("C:\\Users\\ Desktop\\mRNAprocessing")
library(e1071), library(parallel), library(preprocessCore)
source("CIBERSORT.R")
```

```
data <- read.csv("tpm_merge_clear.csv", check.names = F, row.names = 1)

# Run CIBERSORT
result <- CIBERSORT('LM22.txt', 'data.txt', perm = 1000, QN = F)

# Generate violin plots
library(corrplot), library(ggplot2), library("pheatmap"), library("reshape2"), library("dplyr"),
library(ggpubr)

results <- read.table("CIBERSORT-Results.txt", sep="\t", header=T, check.names=F)
rownames(results) <- results[, 1]
results <- results[, -1]
results$group <- c(rep('HT', 50), rep('NONHT', 279))

# Generate violin plots for immune cell infiltration proportions
a <- reshape2::melt(results, id.vars="group", variable.name="genes", value.name="proportion")
for (i in unique(a$genes)) {
    p <- ggplot(a[a$genes == i, ], aes(x=group, y=proportion, fill=group)) +
        geom_violin(trim=FALSE, scale="area") +
        geom_boxplot(width=0.05) +
        stat_compare_means(method = "wilcox.test") +
```

```
        scale_fill_manual(values=c("#FE817D", "#0070C8")) +
        theme_bw() +
        ylab("Proportion") +
        xlab("Group") +
        ggtitle(i)
    ggsave(file=paste0(i, ".pdf"), p, width=8, height=6)
}
```

1.5 Identification and Functional Enrichment Analysis of Differentially Expressed Genes

```
dge<- calcNormFactors(dge)
v <- voom(dge, design, plot = TRUE,normalize.method = "quantile")
fit <- lmFit(v, design)
contrast.matrix <- makeContrasts(contrasts = c("HT-NONHT"), levels = design)
contrast.matrix <- makeContrasts(HT-NONHT,levels=design)
fit2 <- contrasts.fit(fit, contrast.matrix)
fit2 <- eBayes(fit2)
plotSA (fit2)

tempOutput = topTable(fit2, adjust.method = 'BH',coef=1, n=Inf,sort.by = 'logFC')
tempOutput$ENSEMBL<-rownames(tempOutput)
tempOutput<-na.omit(tempOutput)
upgenes<-tempOutput[(which(tempOutput$adj.P.Val<0.05&tempOutput$logFC>1)),7]
downgenes<-tempOutput[(which(tempOutput$adj.P.Val<0.05 & tempOutput$logFC<(-1))),7]
```

```
tempOutput$threshold[(tempOutput$logFC>=(-1)    &    tempOutput$logFC    <=    1)    &
tempOutput$adj.P.Val < 0.05] <- "non"
tempOutput$threshold[tempOutput$logFC>1 & tempOutput$adj.P.Val < 0.05] <- "up"
tempOutput$threshold[tempOutput$logFC<(-1) & tempOutput$adj.P.Val < 0.05] <-"down"
write.csv(tempOutput,file="tempOutput.csv")


# volcano plot
library("ggplot2")
dif<-tempOutput[(which(tempOutput$adj.P.Val<0.05 )),]
DEGs_volcano<-ggplot(dif,aes(x=logFC,y=-log10(adj.P.Val),colour=threshold))+
  geom_point(size=0.5)+
  xlab("logFC")+ylab("-log10 adjust p-value")+
  scale_color_manual(values =c("up"="red","non"="lightgray","down"="green"))+
  geom_vline(xintercept=c(-1,1), lty = 3,colour="black")+
  geom_hline(yintercept =-log10(0.05), lty = 3,color = 'black')+
  theme(legend.title = element_blank(),
  panel.background=element_blank(),
  panel.border=element_rect(fill='transparent', color='black'),)
ggsave("DEGs_volcano.tiff",DEGs_volcano)
ggsave("DEGs_volcano.pdf",DEGs_volcano)
#heat
library(pheatmap)
```

```r
tpm<-read.csv("tpm_merge_clear.csv",row.names = 1,check.names = F)

degnames<-scan(file="degnames.txt",what = "")

deg_tpm<-tpm[rownames(tpm)%in%degnames,]

data1<-log2(deg_tpm+0.01)

annotation_col<-data.frame(Type=factor(c(rep("HT",49),rep("nonHT",253))))

rownames(annotation_col) = colnames(data1)

colnames(annotation_col)=c("Group")

upnames<-scan(file="upnames.txt",what = "")

downnames<-scan(file="downnames.txt",what = "")

up<-data1[rownames(data1)%in%upnames,]

down<-data1[rownames(data1)%in%downnames,]

data2<-rbind(up,down)

annotation_row<-data.frame(Type=factor(c(rep("up",369),rep("down",41))))

rownames(annotation_row) = rownames(data2)

colnames(annotation_row)=c("Type")

DEGs_heatmap<-pheatmap(data2,scale="none",treeheight_row = 0,

  show_colnames = F,show_rownames = F,

  cluster_rows = T,cluster_cols = F,

  annotation_col = annotation_col,

  #annotation_row = annotation_row,

  color = colorRampPalette(c("green","black","red"))(500),

  angle_col = 0,
```

```
  #cellheight=50,#cellwidth=5,cellheight=5,
  fontsize=8,
  width = 10)
ggsave("DEGs_heatmap.tiff",DEGs_heatmap)


library(factoextra)
group_list<-c(rep("HT",49),rep("nonHT",253))
exp<-log2(deg_tpm+1)
colnames(exp)<-group_list
exp<-t(exp)
exp<-as.data.frame(exp)
res.pca <- prcomp(exp, scale = TRUE)
tiff("DEGs_PCA.tiff")
fviz_pca_ind(res.pca,
  geom.ind = "point",
  col.ind = group_list,
  palette = c("#00AFBB", "#FC4E07"),
  addEllipses = T, # Concentration ellipses
  ellipse.type = "confidence",
  legend.title = "Group",# Legend
  repel = TRUE)
dev.off()
```

```
# GO-KEGG

setwd("C:\\Users \\Desktop\\mRNAprocessing\\3.GOKEGG")

BiocManager::install("org.Hs.eg.db")

install.packages("colorspace")

install.packages("stringi")

BiocManager::install("DOSE")

BiocManager::install("clusterProfiler")

BiocManager::install("enrichplot")

library("clusterProfiler")

library("org.Hs.eg.db")

library("enrichplot")

library("ggplot2")

library(GOplot)

genelist<-EC$genelist#circle_dat

david<-EC$david



degs=read.csv("degs.csv",check.names=F,row.names=1)

geneid=as.vector(degs[,7])

entrezIDs <- mget(geneid, org.Hs.egSYMBOL2EG, ifnotfound=NA)

entrezIDs <- as.character(entrezIDs)

out=cbind(degs,entrezID=entrezIDs)

#out=out[is.na(out[,"entrezID"])==F,]
```

```
write.csv(out,"out_na.csv")

out=read.csv("out.csv",check.names=F,row.names=1)

genes<-out[,c(7,1,2,3,4,5,6)]

colnames(genes)<-colnames(genelist)

#GO

gene=out$entrezID

geneFC=out$logFC

names(geneFC)=gene


ego_all <- enrichGO(OrgDb=org.Hs.eg.db,

gene = gene,

pvalueCutoff = 0.05,

pAdjustMethod = 'BH',

qvalueCutoff =0.05,

ont = "ALL",

readable=T)

ego_result<-ego_all@result

ego_result$geneID<-gsub("/", ",", ego_result$geneID)

ego_result<-ego_result[,c(1,2,3,9,7)]#322

colnames(ego_result)<-colnames(david)

write.csv(ego_result,"deg_go_output.csv",row.names=F)

bp<-as.data.frame(ego_result[which(ego_result$Category %in% "BP"),])[1:20,]
```

```
cc<- as.data.frame(ego_result[which(ego_result$Category %in% "CC"),])[1:8,]

mf<- as.data.frame(ego_result[which(ego_result$Category %in% "MF"),])[1:12,]

ego_result_all<-rbind(bp,cc,mf)#40

circ <- circle_dat(ego_result_all , genes)

#BP/CC/MF

#CC lfc.col=c("red","blue")，BP/MF lfc.col=c("red")


pdf("deg_bp.pdf",width = 15,height =8)

GOCircle(circle_dat(bp, genes),nsub = 8,label.size = 7,zsc.col =
c("#FF3333","white","#6699FF"),lfc.col=c("#FF3333","#3399FF"),table.legend = F )+

    theme_bw() +

    theme(    axis.text = element_blank(),

        axis.ticks = element_blank(),

        axis.title = element_blank(),

        panel.border = element_blank(),

        panel.grid.major = element_blank(),

        panel.grid.minor = element_blank()    )

dev.off()

#KEGG enrichment


ekegg<-enrichKEGG(gene= gene,organism = "hsa", qvalueCutoff = 1,pvalueCutoff =
1,pAdjustMethod = "BH")
```

```
ekegg_result<-ekegg@result

ekegg_result<-ekegg_result[ekegg_result$p.adjust <0.05,]

#ekegg_result<-ekegg_result[1:20,]

x=ekegg_result$GeneRatio

y=factor(ekegg_result$Description,levels = ekegg_result$Description)

p = ggplot(ekegg_result,aes(x,y))+ geom_point(aes(size=Count,color=p.adjust))+

scale_color_gradient(low = "SpringGreen", high = "DeepPink")+ labs(color=expression(p.adjust),

size="Count",x="GeneRatio",y="")+

theme_bw() +

theme(legend.text = element_text(size = 16),

legend.title = element_text(size = 14),

axis.text.y = element_text(size = 14),)

ggsave(p,file="deg_KEGG.pdf",width = 12,height =8)

#ggsave(p,file="deg_KEGG.tiff",width = 12,height = 8)

dev.off()

write.csv(ekegg_result,"deg_kegg.csv",row.names = F)

# GSEA

setwd("C:\\Users \\Desktop\\mRNAprocessing\\4.GSEA")

degs=read.csv("degs.csv",check.names=F,row.names=1)

library(clusterProfiler)

library(org.Hs.eg.db)

library(GOplot)
```

```
library(enrichplot)

library(data.table)

geneList<-degs$logFC

names(geneList)<-degs$ENSEMBL

geneList<-sort(geneList,decreasing = T)

#GSEA

#ALL-GO

go_gse_result<-gseGO(geneList=geneList,OrgDb=org.Hs.eg.db,keyType = "SYMBOL",

ont="ALL",minGSSize=10, eps = 0,maxGSSize=1000,nPermSimple = 100000,pvalueCutoff =

1,verbose=F)#634

result<-go_gse_result@result[which(go_gse_result@result$p.adjust<0.05),]#441

go_gse_result@result<-result

write.csv(go_gse_result@result,"go_ges.csv",row.names = F)

#BP

gsebp_result<-gseGO(geneList=geneList,OrgDb=org.Hs.eg.db,keyType = "SYMBOL",

  ont="BP",minGSSize=10, eps = 0,maxGSSize=1000,nPermSimple = 100000,pvalueCutoff =

1,verbose=F)#500

result1<-gsebp_result@result[which(gsebp_result@result$p.adjust<0.05),]#338

gsebp_result@result<-result1

write.csv(gsebp_result@result,"bp_ges.csv",row.names = F)

p1<-gseaplot2(gsebp_result, 1:5, base_size = 18)#展示前 5,展示 P 值 pvalue_table = TRUE

ggsave(p1,file="gsea_BP.pdf",width = 15,height = 8)
```

```
#CC
gsecc_result<-gseGO(geneList=geneList,OrgDb=org.Hs.eg.db,keyType = "SYMBOL",
  ont="CC",minGSSize=10, eps = 0,maxGSSize=1000,nPermSimple = 100000,pvalueCutoff =
1,verbose=F)#500
result2<-gsecc_result@result[which(gsecc_result@result$p.adjust<0.05),]
gsecc_result@result<-result2
write.csv(gsecc_result@result,"cc_ges.csv",row.names = F)
p2<-gseaplot2(gsecc_result, 1:5, base_size = 18)
ggsave(p2,file="gsea_CC.pdf",width = 15,height = 8)
#MF
gsemf_result<-gseGO(geneList=geneList,OrgDb=org.Hs.eg.db,keyType = "SYMBOL",
  ont="MF",minGSSize=10, eps = 0,maxGSSize=1000,nPermSimple = 100000,pvalueCutoff =
1,verbose=F)#500
result3<-gsemf_result@result[which(gsemf_result@result$p.adjust<0.05),]
gsemf_result@result<-result3
write.csv(gsemf_result@result,"mf_ges.csv",row.names = F)
p3<-gseaplot2(gsemf_result, 1:5, base_size = 18)
ggsave(p3,file="gsea_MF.pdf",width = 15,height = 8)
#GSEA-KEGG
id=read.csv("out.csv",check.names=F,row.names=1)
geneList<-id$logFC
names(geneList)<-id$entrezID
```

```
geneList<-sort(geneList,decreasing = T)

gsekegg_result<-gseKEGG(geneList=geneList,organism = "hsa",keyType =
"kegg",minGSSize=10, ,maxGSSize=1000,nPermSimple = 100000,pvalueCutoff =
1,verbose=F)#8

result4<-gsekegg_result@result[which(gsekegg_result@result$p.adjust<0.05),]

gsekegg_result@result<-result4

write.csv(gsekegg_result@result,"kegg_ges.csv",row.names = F)

p4<-gseaplot2(gsekegg_result,1:3, base_size = 18)

ggsave(p4,file="gsea_KEGG.pdf",width = 15,height = 8)
```
